# Supplementary material for: The Fecal Redox Potential in Healthy and Diarrheal Pigs and Their Correlation with Microbiota
Source: Antioxidants (Basel). 2024 Jan 12;13(1):96. doi: 10.3390/antiox13010096 (PMC10812559; doi:10.3390/antiox13010096)
Supplement: Supplementary file 1 [file antioxidants-13-00096-s001.zip › antioxidants-2759517-supplementary.pdf]

Supplementary Table S1. Dietary composition and nutrient levels for weaned piglets

Ingredient composition (%)	Content	Nutrients levels (%)	Content
Puffed corn flour	24.0	Crude protein	18.00
Broken rice noodles	16.8	Crude fibre	6.00
Fish meal	3.0	Ash	8.00
Puffing of soybean	5.0	Ca	0.86
Soybean oil	0.65	Digestible P	0.55
Fermented soybean meal	8.0	Lysine	1.5
Stone powder	0.8		
Calcium dihydrogen phosphate	0.74		
Flour	5.00		
Sugar	3.00		
Glucose	4.0		
Milk powder	10.0		
Low protein whey powder	10.0		
Soy protein concentrate	4.0		
High nucleotide yeast hydrolysate	1.0		
4% Suckling pig premix[1]	4.0		
Total	100		

[1] The premix provided the following per kg of diets: VA 11 000 IU; VD_3 1 000 IU; VE 16 IU; VK $_1$1 mg; VB_1 0.6 mg; VB_2 0.6 mg; VB_6 1.5 mg; VB_{12} 0.03 mg; VB_4 800 mg; biotin 6 mg; nicotinic acid 10 mg; folic acid 0.8 mg; Fe 165 mg; Zn 165 mg; Cu 16.5 mg; Mn 30 mg; Co 0.15 mg; I 0.25 mg; Se 0.25 mg.

Supplementary Table S2. Dietary composition and nutrient levels for growth and finishing pigs

Ingredient composition (%)	Growth period[3]	Finishing period[3]	Nutrients levels (%)	Growth period	Finishing period
Corn	70.0	80.0	Digestive energy[2]	14.60	14.20
Soybean meal	18.0	16.83	Crude protein	16	14
Wheat bran	6.50	0.00	Lysine	1.23	0.73
Soybean oil	1.90	0.30	Methionine/cystine	0.70	0.42
Lysine	0.69	0.22	Threonine	0.79	0.47
Methionine	0.24	0.00	Tryptophan	0.22	0.13
Threonine	0.30	0.05			
Tryptophan	0.07	0.00			
Calcium hydrogen phosphate	0.45	0.65			
Stone powder	0.5	0.5			
Salt	0.3	0.3			
Multivitamins and minerals[1]	0.23	0.23			
Choline chloride (50%)	0.12	0.12			
Zeolite powder	0.6	0.7			
Antioxidant and antifungal agent	0.1	0.1			
Total	100.0	100.0			

[1]The multivitamins and minerals supply per kg diet as follows:VA 11 000IU, VD_3 1 000 IU, VE 16 IU, VK_1 1 mg, VB_1 0.6 mg, VB_2 0.6 mg, VB_3 10 mg, VB_{12} 0.03 mg, VB_9 0.8 mg, VB_6 1.5 mg, Fe 165 mg, Zn 165 mg, Cu 16.5 mg, Mn 30 mg, Co 0.15 mg, I 0.25 mg, Se 0.26 mg. [2]The unit of digestive energy is MJ/kg.

[3]Growth period 85-105 days of age, finishing period 105-162 days of age.
